# The Role of Low-Level Laser Therapy in Bone Healing: Systematic Review

**DOI:** 10.3390/ijms24087094

**Published:** 2023-04-12

**Authors:** Micaela Berni, Alice Maria Brancato, Camilla Torriani, Valentina Bina, Salvatore Annunziata, Elena Cornella, Michelangelo Trucchi, Eugenio Jannelli, Mario Mosconi, Giulia Gastaldi, Laura Caliogna, Federico Alberto Grassi, Gianluigi Pasta

**Affiliations:** 1 Orthopedics and Traumatology Clinic, IRCCS Policlinico San Matteo Foundation, 27100 Pavia, Italy; 2 Department of Molecular Medicine, University of Pavia, 27100 Pavia, Italy; 3 Centre for Health Technologies, University of Pavia, 27100 Pavia, Italy; 4 Department of Clinical, Surgical, Diagnostic and Pediatric Sciences, University of Pavia, 27100 Pavia, Italy

**Keywords:** low-level laser therapy (LLLT), phototherapy, photo-biomodulation, bone regeneration, bone healing, bone fracture healing, orthopedics treatments

## Abstract

Low-level laser therapy (LLLT) is a treatment that is increasingly used in orthopedics practices. In vivo and in vitro studies have shown that low-level laser therapy (LLLT) promotes angiogenesis, fracture healing and osteogenic differentiation of stem cells. However, the underlying mechanisms during bone formation remain largely unknown. Factors such as wavelength, energy density, irradiation and frequency of LLLT can influence the cellular mechanisms. Moreover, the effects of LLLT are different according to cell types treated. This review aims to summarize the current knowledge of the molecular pathways activated by LLLT and its effects on the bone healing process. A better understanding of the cellular mechanisms activated by LLLT can improve its clinical application.

## 1. Introduction

Light Amplification by Stimulated Emission of Radiation, commonly known by the acronym LASER, is a technological device developed in the 1960s capable of emitting a light beam with precise characteristics of monochromatic, coherence, unidirectionality, collimance and radiance, which make it a versatile device for various daily areas, including industrial production, telecommunications, scientific research and in the medical area. In particular, in the latter field, the laser has two main applications: surgery and biostimulation [1]. In the surgical branch, the use of lasers with a power of over 500 mW, called high-intensity lasers (HIL), is preferred; these use heat to induce photothermal damage of various intensities [2]. In the biostimulation field, low-level lasers (LLL) are used, with a power lower than 500 mW and an emission spectrum between red and in the near-infrared region because these lasers do not produce heat during their work [3]. Since the 1980s, they have been studied for applications in the biomedical field, but unfortunately, their mechanisms are still unclear [4]. Several studies of the use of low-level lasers as clinical therapy (LLLT) have been reported in the literature since they would seem to increase the speed of mitochondrial electron transport by stimulating the production of ATP by the mitochondria, reduce oxidative stress by modulating the reactive species of the oxygen and induce several transcription factors, including AP-1, p53, NF-kB and HIF, which allow extracellular matrix deposition, and activation of anti-inflammatory and anti-apoptotic pathways. This effect manifests itself in the clinical setting with a reduction in pain and inflammation, an improvement in tissue repair, the promotion of regeneration of various tissues and nerves, and the prevention of tissue damage [5,6,7]. In vitro studies have shown that the application of LLLT can influence several cellular processes and molecular pathways such as the alteration of deoxyribonucleic acid (DNA) synthesis, the gene expression, the increase of cytokines production and the cellular proliferation [8]. Moreover, interesting in vitro studies have shown that LLLT can stimulate proliferation of several types of cells such as fibroblasts, endothelial cells and keratinocytes, and it can also modulate the production of different inflammation factors [9]. 

Bone regeneration is an optimal physiological process that has four main overlapping phases: the inflammatory phase, the angio-mesenchymal phase, the bone formation phase and the bone remodeling phase. Bone infections, major pathological fractures and systemic diseases can interfere in this process, up to causing failure of repair [10]. In recent decades, many efforts have been made to understand the regeneration of musculoskeletal tissue and to identify clinical approaches capable of stimulating and accelerating this process. The approaches that play a crucial role in this process are: chemical treatments, physical stimulation including mechanical forces, the use of ultrasound and shock waves and Scaffold or Electrical or Electromagnetic stimulation, and photostimulation [11]. Several studies present in the literature confirm a positive influence of laser therapy in the bone regeneration process; in particular, they show a possible influence of photostimulation at the level of the molecular mechanisms present in the phase of the process. Since there are different studies in the literature that report information, sometimes even conflicting, on the role of laser therapy in bone regeneration, the purpose of this review is to summarize the main evidence reported in literature, clarify the actual role of photostimulation at the level of the molecular pathway, and identify a possible clinical use of LLLT.

## 2. Materials and Methods

This report provides an account of the review of the available literature conducted in order to achieve the aim of the research. Its reporting is an adaptation of the Preferred Reporting Items for Systematic Reviews and Meta-Analyses (PRISMA) guidelines [12].

- Identifying the research question

The research question identified for the literature review was first of all to summarize the current knowledge of the molecular pathways activated by LLLT and its effects on the bone healing process.

- Identifying relevant studies

A literature search was conducted in order to find all relevant studies on the topic. These were identified by means of diverse sources.

- Electronic database search

The following electronic databases were searched, taking into consideration the chronological span between 2002 and 2022: PubMed and Embase

The research strategy was carefully designed, in order to retrieve the most relevant results. Due to the specificity of the two databases employed, for each one a different search string was built (1: PubMed search string; 2: Embase search string). In brackets, the number of the results is provided).

1. ((“low level light therapy” [MeSH Terms] OR (“low level” [All Fields] AND “light” [All Fields] AND “therapy” [All Fields]) OR “low level light therapy” [All Fields] OR (“low” [All Fields] AND “level” [All Fields] AND “laser” [All Fields] AND “therapy” [All Fields]) OR “low level laser therapy” [All Fields]) AND (“bone and bones” [MeSH Terms] OR (“bone” [All Fields] AND “bones” [All Fields]) OR “bone and bones” [All Fields] OR “bone” [All Fields] OR (“mesenchymal stem cells” [MeSH Terms] OR (“mesenchymal” [All Fields] AND “stem” [All Fields] AND “cells” [All Fields]) OR “mesenchymal stem cells” [All Fields]))) AND ((fft [Filter]) AND (English [Filter]) AND (2002: 2022 [pdat])) [953];
2. (“low level laser therapy”/exp OR “lllt (low level laser therapy)” OR “low-intensity (therapeutic) laser therapy (lilt)” OR “laser biostimulation” OR “laser therapy, low-level” OR “low energy laser therapy” OR “low energy laser treatment” OR “low intensity laser therapy” OR “low intensity laser treatment” OR “low level laser therapy” OR “low level laser treatment” OR “low level light therapy” OR “low power laser therapy” OR “low power laser treatment” OR “low-level laser therapy” OR “low-level laser therapy (lllt)” OR “low-level light therapy” OR “photo biomodulation therapy” OR “photo-bio-modulation therapy” OR “photo-biomodulation (pbm) therapy” OR “photo-biomodulation therapy (pbmt)” OR “photobiomodulation (pbm) therapy” OR “photobiomodulation therapy” OR “photobiomodulation therapy (pbm)” OR “photobiomodulation therapy (pbmt)” OR “soft laser therapy” OR “therapeutic laser therapy”) AND (“bone”/exp OR “mesenchymal stem cells”) AND [2002–2022]/py AND [english]/lim [1069].

- Other sources

Twenty studies were also included, starting from a review excluded from the analysis, since only primary studies were considered according to the inclusion criteria. These articles were regarded to be relevant, even though they were not identified through the search strings.

- Study inclusion

Starting from the research question, inclusion and exclusion criteria for the objective selection of the studies identified were defined. Only studies published in the English language between 2002 and 2022 were eligible for inclusion. Titles and abstracts and full texts were screened by the research team—i.e., two authors performed the study selection and the data extraction independently, and all disagreements were discussed between the authors.

- Data extraction

A standardized data extraction sheet was prepared, where main information on the studies was collected (e.g., first author’s name, study title, publication year and DOI).

- Study selection

**Figure 1:**
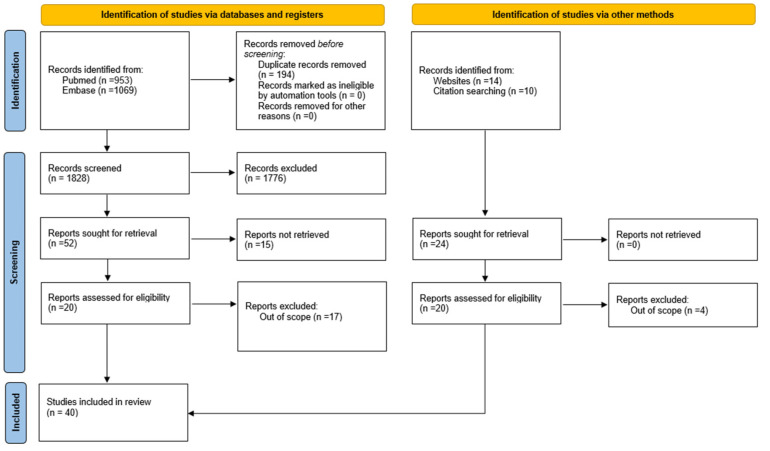
PRISMA flow diagram that included searches of databases, registers and other sources [12].

Via the comprehensive literature search, forty studies were included in this literature review; twenty of them were identified via database searches and twenty via websites or citation searching (Figure 1). Figure 1 shows the process of study selection in detail, covering the number of search records retrieved from the two database searches (*n* = 2022) and all other searches (*n* = 24), the number of screened titles/abstracts (*n* = 1828), the number of finally included studies (*n* = 40).

## 3. Results

### 3.1. Low-Level Laser Therapy (LLLT)

Laser therapy is a therapeutic approach widely used in various areas of the medical field, including the biostimulation of different tissues. In this case, the use of a laser with a power lower than 500 mW and an emission spectrum between 500 and 1000 nm is able to interfere with the molecular pathways promoting the regeneration and repair of various tissues and preventing any damage.

LLLT has also recently been used in the orthopedic field to promote the bone healing process; in particular, the laser used is based on low-density energy, which uses non-thermal radiant photons from coherent (laser) or non-coherent (LED) light sources. To have effective treatment in promoting bone regeneration, LLLT must comply with specific dosimetric parameters; unfortunately, the evidence in the literature is conflicting in highlighting precise parameters, so it was only possible to identify a range of values that would seem optimal (Table 1), below which the treatment is not very effective while above it tends to lead to tissue damage [13].

Currently, the most accredited theories hypothesize that LLLT influences various biological activities: It promotes the enzymatic activity of cytochrome c oxidase (CCO) through the photodissociation of inhibitory nitric oxide from CCO, with consequent in-creased mitochondrial respiration and production of adenosine triphosphate (ATP) [14,15]. LLLT also correlates in the modification of the mitochondrial membrane potential, causing the increase in the redox state of the cells with the consequent increase in the oxidation of Fe^2+^, the inhibition of proline hydroxylases (PHDs) and the deregulation of the factor HIF-1α. Furthermore, the increase in ROS leads to the alteration of several signaling pathways, including the cell proliferation, survival, repair and regeneration pathways [5,16,17].

### 3.2. Molecular Pathways LLT-Activated on Bone Healing

Bone regeneration tends to be an optimal physiological process, but in the presence of bone infections, blood insufficiency, bone defects, pathological fractures, and systemic diseases, repair failure is possible [10]. Different clinical approaches have been studied to stimulate and accelerate this process, including physical stimulation, chemical treatment, and photo-stimulation [11]. LLLT is used to promote the bone healing process, which is composed of four main overlapping phases: the inflammatory phase, angio-mesenchymal phase, the bone formation phase and the bone remodeling phase [18]. The inflammatory phase is characterized initially by the recruitment and activation of pro-inflammatory signals and growth factors, including tumor necrosis factor-α (TNFα) and several interleukins, followed by the recruitment of polymorphonuclear neutrophils (PMNs), macrophages and platelets, which together with the bone morphogenetic proteins (BMP), already present in the fracture site, recall mesenchymal stem cells (MSC) [19]. A key modulator of this phase is Wnt, a signaling pathway capable of activating different molecular pathways, including Wnt/ß-catenin (canonical), Wnt/Ca^2+^, Wnt/planar cell polarity, and Wnt/protein kinase (non-canonical) [20]. In the angio-mesenchymal phase, characterized by high levels of VEGF, PDGF and FGF, the invasion and formation of new blood vessels takes place and the transformation of the avascular cartilage matrix into bone tissue is vascularized. In particular, VEGF, a transcription factor expressed by hypertrophic osteoblasts and chondrocytes, promotes both neo-angiogenesis, inducing the formation of new vessels from those already present, and revascularization of the fracture site, aiding cell proliferation and aggregation endothelial stem cells [21]. MSC recruitment marks the initiation of the bone renewal phase, which is characterized by the differentiation of MSCs into osteoblasts or chondrocytes for newly forming bone. The transcription factors that play a crucial role in this phase are: *runx-2* and *osterix*. *Runx-2* affects the early phases of osteoblast differentiation by regulating the expression of different bone matrix genes, while *osterix* intervenes in the regulation of differentiation in the late phases when runx-2 is down regulated. This phase of bone regeneration is characterized by several molecular pathways, including bone morphogenetic protein signaling pathway (BMP), tumor growth factor-β signaling pathway (TGF-β), Phosphoinositide 3-kinase/Akt/Mammal target rapamycin signaling pathway (PI3K/Akt/mTOR) and the mitogen-activated protein kinase pathway (MAPK). At the end of the renewal, mature osteoblasts deposit collagen and calcium at the fracture site, allowing the formation of callus, also called primary bone [22]. The correct formation of the bone callus, which is important for a complete healing of the fracture, is guaranteed by the balance between the osteoblasts and osteoclasts, which respectively allow the formation of new bone or its resorption. This process is regulated by growth hormone (GH), which through direct stimulation (GH) and indirect (stimulation mediated by the hormone IGF-1 insulin-like growth factor) has two main functions: to stimulate the proliferation and activity of osteoblasts to induce the formation of new bone and symmetrically increase the differentiation and activity of osteoclasts, in order to favor bone resorption. Furthermore, GH also has the role of inducing the terminal differentiation of the chondrocytes present in the areas of bone growth [23]. The bone regeneration process is induced by different pro-inflammatory signals including interleukins, TNFα and interferon-γ [24]. Different approaches can stimulate and influence this process, including chemical treatments, physical stimulation (mechanical forces, ultrasound, shock waves, stimulation of different nature), photostimulation and cell therapies. The latter approach, currently considered the most effective, consists of using mesenchymal stem cells to increase the bone regeneration process, as this cell line has excellent self-renewal and pluripotency properties. MSCs are easily obtained from several tissues, including bone marrow, adipose tissue, skin, umbilical cord and placenta, but the best regenerative results have been obtained from bone marrow MSCs. However, this cellular type also has some negative aspects, among which are high variability in cell quality and concentration, due to the different tissue sources from which they were obtained, as well as challenging costs and times. The presence of these limitations in the use of MSCs has led to the search for new treatments to enhance bone regeneration, and among these is the LLLT. Photostimulation would seem to influence several biological activities, including mitochondrial respiration, ATP production and ROS levels in cells, causing a possible alteration of some cellular biological processes [17]. Therefore, several studies have been conducted to understand the effects of LLLT, both in vitro, mainly on BMSC, HUVEC, MSC cells and osteoblasts, and in vivo, especially on rat and rabbit.

Below are some studies, conducted both in vivo and in vitro, showing the possible effects of LLLT.

### 3.3. In Vitro Studies

**Figure 2:**
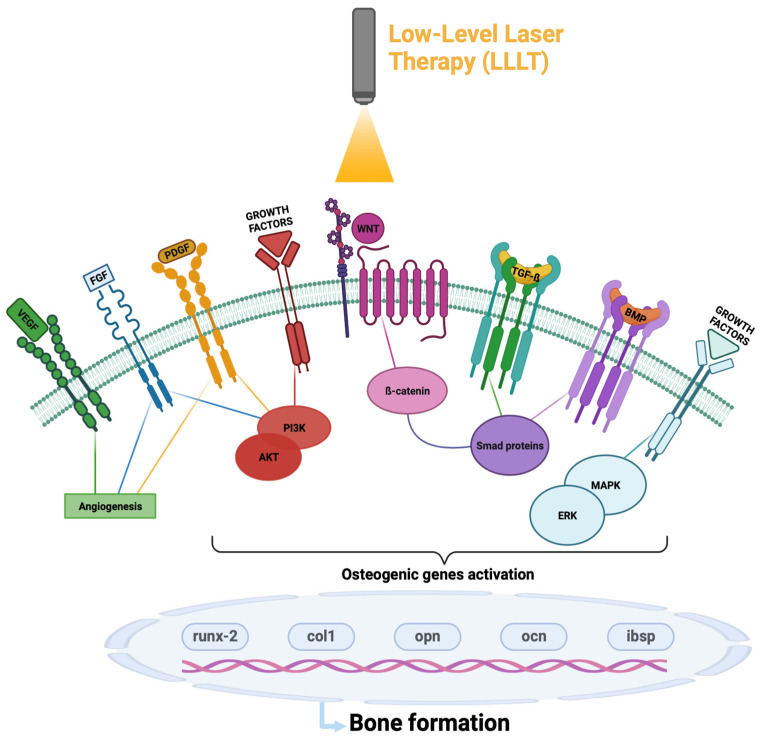
Schematic representation of molecular pathways activated by Low-Level Laser Therapy (LLLT). Abbreviations: VEGF (vascular endothelial growth factor); FGF (Fibroblast Growth Factors); PDGF (Platelet-Derived Growth Factor); TGF-ß (tumor growth factor- ß); BMPs (bone morphogenetic proteins); AKT (AKT Serine/Threonine Kinase 1); PI3K(Phosphatidylinositol-4,5-Bisphosphate 3-Kinase Catalytic Subunit Delta); MAPK (mitogen-activated protein kinase); ERK (extracellular signal-regulated kinase 1/2); SMAD proteins (small mothers against decapentaplegic); *runx*-2 (runt-related transcription factor gene); *col1* (collagen type 1 gene); *ocn* (osteocalcin gene); *opn* (ostepontin gene); *ibsp* (bone sialoprotein gene).

It has been observed that LLLT interacts mainly in the initial phases of bone regeneration process; it stimulates the following signaling pathways (Figure 2 and Table 2):

#### 3.3.1. Inflammatory Phase

- WNT/β-catenin is a signaling pathway that plays a crucial role in the progression of bone regeneration; in particular, its activation stabilizes the cytoplasmic β-catenin, allowing its nuclear translocation and degrades the protein complex formed by axin, adenomatosis polyposis coli (APC), protein phosphatase 2A (PP2A), glycogen synthase kinase 3 (GSK3) and casein kinase 1 α (CK1α) responsible for the destruction of ß-catenin [20]. Once activated and translocated to the nucleus, β-catenin interacts with several DNA-bound proteins, including LEF and TFC, to induce the expression of osteogenic markers and the differentiation of pre-osteoblasts [35]. The inflammatory process negatively affects this signaling pathway, since the presence of pro-inflammatory cytokines, including TNF, stimulates the expression of Dickkopf-related protein 1 (DKK-1), an antagonist of the WNT signaling pathway that prevents its activation [36]. Laser treatment, particularity with a wavelength of 830 nm, increases the translocation of β-catenin from the cytoplasm to the nucleus, increasing the activation of the pathway and accelerating the bone regeneration process [25].
- NF-kB is a transcription factor present in the cytoplasm, which, following the phosphorylation of its inhibitor complex IkBα, can translocate into the nucleus and through gene transcription, promoting the inflammation process. Treatment, with a red laser with a wavelength of 660 nm of adipose-derived stem cells (ADSCs), showed a reduction in phosphorylated IkBα and NF-kB concentration in the nucleus, highlighting the ability of LLLT to negatively regulate the process of inflammation via NF-kB inhibition [27].

#### 3.3.2. Angio-Mesenchymal Phase

- VEGF is one of the main regulators of angiogenesis, able to promote the migration and proliferation of endothelial cells. A subclass of endothelial vessels, termed H-type vessels due to high expression of CD31 and endomucin, positively influence osteogenesis by resisting surrounding osteoprogenitors that express high levels of *osterix*, a promoter of bone formation. It has been reported through the literature that irradiation of human bone marrow mesenteric stem cells with an 808 nm laser increased both VEGF expression and H-type vessel formation, leading to an overall enhancement of the angiogenic process [16,29].
- FGF contributes to the regulation of several important cellular processes; by activating the PI3K-AKT signaling pathway, it inhibits cell apoptosis by promoting its proliferation, while by stimulating the MAPK/ERK pathway it induces osteoblast differentiation. It is also involved in the process of angiogenesis and wound healing, and studies in the literature have shown a correlation between irradiation with a 660 nm laser and an increase in FGF expression [28].
- PDGF is a factor that plays a role in the regulation of the mitogenic, angiogenic and proliferative activity of MSC cells. Thanks to its dimerization and the activation of several signaling proteins including phospholipase C, the kinases Src, PI3-kinase and the phosphatase SHP2, it also recalls different growth factors. A significant increase in this factor was observed when treating MSC cells with a laser with a wavelength of 636 nm [26].
- ROS/HIF-1α pathway activation promotes cell differentiation and endothelial angiogenesis, two key mechanisms of bone repair. Recent studies have shown that the GaAlAs laser treatment with 4.5 J/cm2 of BMSC cells, in simple culture and more significantly if in co-culture with HUVECs, led to activation of the ROS/HIF-1α pathway [16].

#### 3.3.3. Bone Formation Phase

- Runt-related transcription factor (*runx-2*) has a relevant role in the recruitment of MSCs and in their differentiation into osteoblasts; it also induces the expression of the bone matrix genes collagen type 1 (*col1a1*), osteopontin (*opn*), osteocalcin (*ibsp*) and osteocalcin (*ocn*) [16,35]. Studies in the literature have shown that laser therapy directs MSCs towards osteogenic differentiation versus adipogenic differentiation [25]. Moreover, an increase in BMSC proliferation and differentiation in osteoblasts was observed after irradiation with a GaAlAs diode laser with a wavelength of 810 nm and an energy density between 2 and 4 J/cm2 [30].
- BMP/TGF-β has a key role in the osteogenic process; in particular, TGF- β plays a crucial role in osteoblast proliferation, while BMP has a remarkable ability in bone formation as well as being fundamental in the maturation process of osteogenesis [23]. In the literature, different studies reported a correlation between LLLT and the increase in TGF- β and BMP expression. In particular, it was observed that the irradiation of osteoblast-like cells (human osteosarcoma cell line MG-63) with LLLT led to an increase in BMP [31] and promoted osteogenic differentiation through the activation of ROS, which in turn activated TGF-β 1 [16]. This correlation has also been found in hypoxic conditions by treating osteoblasts with a GaAlAs laser [32].
- The PI3K/Akt/mTOR signaling pathway, thanks to the activation cascade of its protein effectors, is one of the main pathways involved in cell survival, regulating cell cycle, motility, apoptosis, metabolism and cell differentiation, as well as the mechanisms of transcription and translation. It would also seem to have a correlation with bone regeneration, and in fact there are some studies in the literature showing a possible involvement in bone formation and remodeling [23]. Through these studies, it was observed that exposure to a laser treatment with a wavelength of 650 nm and an energy density between 1 and 4 J/cm2 of the HUVECs cell line increased the phosphorylation of PI3K, AKT and mTOR, allowing greater activation and functionality of the signaling pathway [33]. An interesting study confirmed the role of LLLT on increasing the Akt expression levels and its activated phosphorylated form on osteoblasts irradiated with 635 nm as compared to untreated cells [34].
- The MAPK kinase pathway plays an important role in signal transduction by controlling several cellular processes, including cell proliferation and differentiation as well as responses to cellular stress. Furthermore, it also regulates bone formation, inducing osteogenic differentiation and controlling the vitality and functions of osteoblasts. In particular, the use of a laser with a wavelength of 910 nm on the MC3T3-E1 cell line allowed an increase in ERK phosphorylation, increasing the activity of the pathway [11].

### 3.4. In Vivo Studies

In the literature, it has been observed also in vivo that LLLT has a relevant role in bone repair. In vivo studies were conducted mainly on rats and rabbits, and they showed a significantly accelerated bone healing process after LLLT exposure (Table 3).

Consistent with the results obtained in vitro, in vivo studies have confirmed that laser therapy facilitates the early stages of bone regeneration [41]. Sixty rats that had irradiation with an 830 nm laser showed a modulation of the inflammatory process, with a deregulation of the pro-inflammatory interleukin IL-1, IL-6, IL-8 and IL-18, and anticipated the repair of the bone defect [37]. In addition, the angio-mesenchymal phase showed improvements after laser treatment, with greater stimulation of angiogenic genes in damaged tissues; in particular, the irradiation at 830 nm up-regulates the expression of VEGF [37], while the use of a laser at 1064 nm increased the expression of PDGF and FGF [38]. In vivo studies also confirmed more osseointegration of bone implants after treatment with laser therapy, with an increase in the expression of different bone formation marker proteins, including BMP2 and OCN, after irradiation with an 808 nm GaAlAs laser [13].

Currently, in the literature, there is only a little evidence about an effective beneficial effect after the LLLT treatment on patients that agrees on the efficacy of laser therapy, mainly in the early stages of bone regeneration. These studies showed an increase in bone formation and mineralization and an acceleration in osteoblast differentiation, after having subjected patients to laser treatment with a wavelength of 830 nm and an energy density of 16 J/cm^2^ [39]. A marked increase in bone regeneration was also confirmed by a double-blind clinical trial conducted on patients after the surgically assisted rapid maxillary expansion that has shown an improvement in bone regeneration and a decrease in complications thanks to a postoperative treatment with GaAlAs laser with a wavelength of 660 nm and an energy density of 2.7 J/cm^2^ [40].

## 4. Discussion

Laser therapy is based on the use of a technological device capable of emitting a light beam with precise characteristics that make it versatile for various fields, including the medical one. The choice of a laser with a power lower than 500 mW and an emission spectrum between red and infrared allows for a therapy that does not generate heat, which is potentially harmful to the tissues, capable of stimulating and promoting the bone healing process.

Various studies conducted both in vivo and in vitro, have shown that the effect of laser treatment increased the bone regeneration process only in the first weeks after the fracture (2/4 weeks), while after about 60 days there was no advantage observed over controls [42], indicating that LLLT is effective only in the early stages of the process. Researches conducted in the literature did not reveal specific physical parameters that make LLLT optimal but provided a range of values, outside of which the treatment is probably ineffective. These results suggest that low-level laser stimulation activates a common conserved response mechanism to physical stimuli. Furthermore, a good influence of LLLT on regeneration was observed regardless of the cell line considered. Bone regeneration is an optimal physiological process that, however, can present problems and fail in the presence of pathological fractures and systemic diseases. Despite having four major overlapping phases, results in the literature seem to indicate that LLLT affects only the molecular pathways involved in the first three phases, in particular by activating signaling pathways involved in the angio-mesenchymal phase and the bone formation phase, and by inhibiting that characteristic of the inflammatory phase. The inflammatory response, triggered by blood vessels and bone breakdown, plays an important role in bone healing by enabling the recruitment of cells required for tissue repair and regeneration [20]. In the literature, it is reported that the application of LLLT can help modulate the inflammatory response: in vitro, due to both WNT activation and inhibition of NF-kβ signaling [43], and in vivo, with a deregulation of the inflammatory pro-interleukin IL-1, IL-6, IL-8 and IL-18, and has anti-patent bone defect repair [29]. These studies would seem to indicate a negative regulation of stimuli by LLLT. As regards the angio-mesenchymal phase, characterized by the formation of new blood vessels and the transformation of the avascular cartilaginous matrix into vascularized bone tissue, several studies have reported that LLLT treatments have been able to induce an increase in VEGF, FGF and PDGF in stem cells (hBMSC, hMSC and hOB), as well as the production of termed H-type vessels, a subcategory of endothelial vessels that exhibit high levels of CD31. These results were also found in in vivo studies conducted on rats. Activation of these signaling pathways promoted proliferation, osteogenic differentiation and angiogenesis [16,26,29]. The recruitment of MSCs and their differentiation into osteoblasts characterizes the bone formation phase; this phase of the process also seems to be positively influenced by laser treatment. Both in vitro and in vivo studies confirm this hypothesis; in fact, an increase in BMP and TGF-beta signaling has been reported, with the consequent promotion of osteogenic differentiation, by exposing both stem cells (hMSC, human osteosarcoma cell line MG-63), in rats. However, as far as the bone remodeling phase is concerned, the studies conducted so far in the literature have not highlighted a possible role of laser therapy; only one recent in vitro study showed a possible effect on increasing bone mineralization by irradiation of human osteoblasts with different types of LLLT [44]. This evidence is supported by few relevant data; therefore, it would be appropriate to conduct further investigations to validate this hypothesis.

## 5. Conclusions

All the data reported in the literature show different clinical applications of LLLT but do not provide accurate knowledge on the stimulation of cellular responses due to the wide difference in dosimetry parameters (wavelength, power density, energy, irradiation time, frequency repetition and distance from the sample) used in the different studies. Therefore, it will be necessary to carry out more accurate analyses such as investigating in vivo cell survival, apoptosis, epigenetic changes and stress responses to have a broader view of the entire cellular context and the physiopathology of tissues. However, a better understanding of the in vitro effects of LLLT on biological systems would be a golden opportunity to predict in vivo outcomes. Due to the lack of standardized experimental guidelines and the great heterogeneity of the LLLT dosimetry parameters used both in vitro and in vivo, the controlled trials produced data that were not comparable and were inconclusive.

## Figures and Tables

**Table 1:** Dosimetry parameters of LED device observed in the literature.

Parameter	LED Range
Wavelength (nm)	500–1000
Power density (J/cm^2^)	0.5–30
Energy (mW)	5–100
Irradiation time (s)	3–1440
Repetition rate (d)	1–60
Distance from sample (cm)	0–14

**Table 2:** Summary of in vitro LLLT studies.

Laser Wavelength	Laser Power Density	Target	Effects	Bone Phase Target	Ref.
830 nm	7.64 J/cm^2^	BMSCs	WNT-ß-catenin activation	Inflammatory phase	[25]
636 nm	5 J/cm^2^	MSCs	PDGF increase	Angio-mesenchymal phase	[26]
660 nm	4–8 J/cm^2^	ADSCs	NF-kB inhibition	Inflammatory phase	[27]
660 nm	6 J/cm^2^	ADSCs	FGF increase	Angio-mesenchymal phase	[28]
808 nm	4.5 J/cm^2^	BMSC	VEGF increase	Angio-mesenchymal phase	[16,29]
808 nm	4.5 J/cm^2^	Co-culture of BMSC and HUVECs	ROS/HIF-1α activation	Angio-mesenchymal phase	[16]
810 nm	2–4 J/cm^2^	BMSC	ALP activity increase	Bone formation phase	[30]
940 nm	NA	MG-63 cell line	BMP increase	Bone formation phase	[31]
808 nm	1.2, 2.4, and 3.6 J/cm^2^	Human fetal osteoblasts	BMP crease	Bone formation phase	[32]
650 nm	1–4 J/cm^2^	HUVEC	PI3K/Akt/mTOR activation	Bone formation phase	[33]
635 nm	0,4 J/cm^2^	Human osteoblast	PI3K/Akt/mTOR activation	Bone formation phase	[34]
910 nm	2.85 J/cm^2^	MC3T3-E1	MAPK kinase pathway activation	Bone formation phase	[11]

**Table 3:** Summary of in vivo LLLT studies.

Laser Wavelength	Laser Power Density	Target	Effects	Bone Phase Target	Ref.
830 nm	NA	rats	Decrease of pro-inflammatory interleukins (IL-1, IL6, IL8, IL18)	Inflammation phase	[37]
830 nm	NA	rats	VEGF increase	Angio-mesenchymal phase	[37]
1064 nm	8 J/cm2	rats	PDGF and FGF increase	Angio-mesenchymal phase	[38]
808 nm	354 J/cm2	rats	BMP2, OCN and ALP increase	Bone formation	[13]
830 nm	16 J/cm2	Human periodontal infra-bony defects	Radiological evidence	Bone formation	[39]
660 nm	2.7 J/cm2	Human surgical disjunction of the maxilla	Accelerating of the repair process.	Bone formation	[40]

## Data Availability

https://pubmed.ncbi.nlm.nih.gov (accessed on 6 February 2023); https://www.embase.com (accessed on 6 February 2023).

## Abbreviations

ADSCs	Adipose-derived stem cells
ATP	Adenosine triphosphate
BMP	Bone morphogenetic proteins
BMSC	Bone marrow Stem Cells
CCO	Cytochrome c oxidase
col1a1	Collagen type I gene
DKK-1	Dickkopf-Related Protein 1
FGF	Fibroblast Growth Factors
HUVEC	Human Umbilical Vein Endothelial Cells
ibsp	Bone sialoprotein 2 gene
IkBα	Inhibitors of NF-kB
LLLT	Low-level laser therapy
MSC	Mesenchymal stem cells
NF-kB	Nuclear Factor kappa B
ocn	Osteocalcin gene
opn	Osteopontin gene
PDGF	Platelet-Derived Growth Factor
PMNs	Polymorphonuclear neutrophils
Runx-2	Runt-related transcription factor gene
TGF-ß	Tumor growth factor- ß
TNFα	Tumor necrosis factor-α
VEGF	Vascular Endothelial Growth Factor
AKT	AKT Serine/Threonine Kinase 1
PI3K	Phosphatidylinositol-4,5-Bisphosphate 3-Kinase Catalytic Subunit Delta
